# The Apoptosis of Liver Cancer Cells Promoted by Curcumin/TPP-CZL Nanomicelles With Mitochondrial Targeting Function

**DOI:** 10.3389/fbioe.2022.804513

**Published:** 2022-02-15

**Authors:** Wanyu Li, Yanan Chen, Kun He, Tianshou Cao, Daibo Song, Huiling Yang, Li Li, Jiantao Lin

**Affiliations:** ^1^ Guangdong Provincial Key Laboratory of Research and Development of Natural Drugs, School of Pharmacy, Guangdong Medical University, Dongguan, China; ^2^ Hepatobiliary Surgery, Zhongshan People’s Hospital, Zhongshan, China

**Keywords:** curcumin, mitochondrial, TPP, targeted, liver cancer

## Abstract

The mitochondrion is one of the most important cellular organelles, and many drugs work by acting on mitochondria. Curcumin (Cur)-induced apoptosis of HepG2 in liver cancer cells is closely related to the function of inhibiting mitochondria. However, the mitochondrion-targeting curcumin delivery system was rarely been reported. It is important to develop a high-efficiency mitochondrion-targeting curcumin vector that can deliver curcumin into mitochondria directly. Here, a special mitochondrion-targeting delivery system based on triphenylphosphine bromide (TPP)-chitosan-g-poly-(N-3-carbobenzyloxy-l-lysine) (CZL) with TPP functional on the surface is designed to perform highly efficient mitochondria-targeting delivery for effective liver cancer cell killing *in vitro*. The TEM images showed that the nanomicelles were spherical; the results of fluorescence test showed that TPP-CZL nanomicelles could promote the cellular uptake of drugs and finally targeted to the mitochondria. The results of cell survival rate and Hoechst staining showed that curcumin/TPP-CZL nanomicelles could promote the apoptosis of liver cancer cells. Curcumin/TPP-CZL nanomicelles could significantly reduce the mitochondrial membrane potential, increase the expression of pro apoptotic protein Bcl-2, and reduce the expression of antiapoptotic Bax protein, and these results were significantly better than curcumin/CZL nanomicelles and curcumin. It is a potential drug delivery system with high efficiency to target mitochondria of liver cancer cells.

## Introduction

Liver cancer is a serious malignant tumor worldwide. It is characterized by a high degree of malignancy, high recurrence and metastasis rate, and bad prognosis (Bray et al., 2018). Surgical operation is the preference for patients with complex liver cancer. However, they usually cannot prevent tumor metastasis, and the operation risk is high, postoperative complications are easy to occur, and the prognosis is not ideal (Yu et al., 2020). The use of cytotoxic drugs in chemotherapy will not only have adverse effects on healthy organs, but also lead to serious adverse side effects such as bleeding and anemia (Lim et al., 2020). In addition, these side effects increase the mortality of cancer patients. Therefore, there is still a lack of effective treatment for liver cancer.

Curcumin is a polyphenol active component extracted from the rhizome of Curcuma longa. In recent years, it has been found that curcumin has excellent antitumor activity (Hanafy, 2021; Nemany et al., 2018), and accumulating studies suggest that it has a significant inhibitory effect on liver cancer. Li et al. found that curcumin (cur)-induced apoptosis of liver carcinoma Bal-7402 and SMMC-7721 cells is closely related to the inhibition of mitochondrial function. Curcumin can affect Bax and Bcl-2, resulting in apoptosis, raised expression of Bax, and lower expression of Bcl-2. Additionally, curcumin could stimulate cytochrome C releasing from mitochondria into cytoplasm, and activate caspase dependent-mitochondrial apoptosis pathway (Li et al., 2012). In this case, it is expected that curcumin will be delivered to the mitochondria as much as possible, which may greatly improve the effect of drugs on tumor cell apoptosis.

**Table:** 

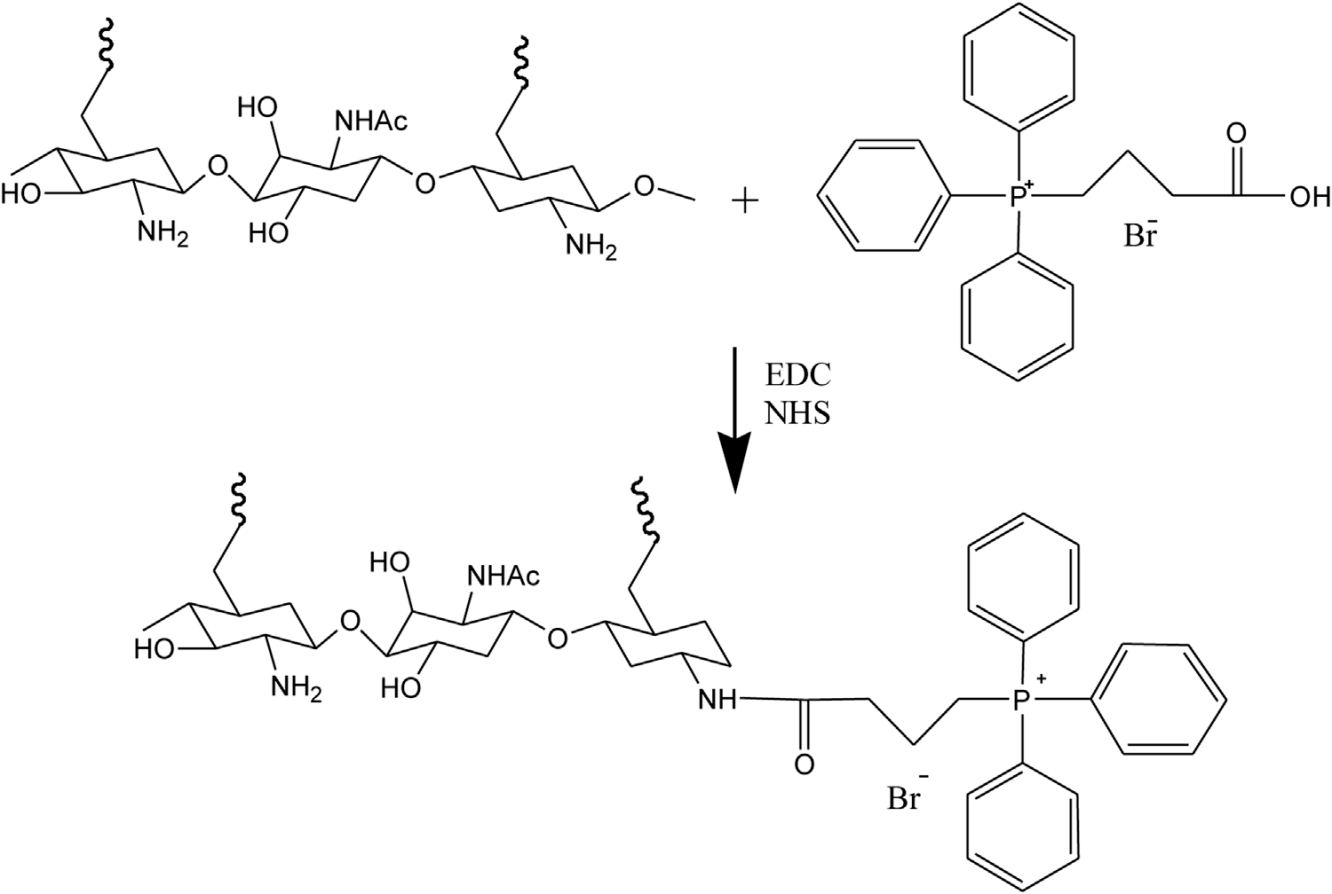

Currently, it is well documented that (3-propylcarboxyl) triphenylphosphinium bromide (TPP) has positive charge property, which can be used to mediate tumor drugs and polymeric drugs targeting into mitochondria (Han et al., 2019; Li et al., 2019; Lee et al., 2021). Therefore, if the vector and TPP were linked together to construct a vector with mitochondrial targeting function, and then loaded with curcumin to prepare curcumin mitochondrial-targeted nanomicelles, it would be possible to deliver more curcumin to the mitochondria of liver cancer cells, give full play to the advantages of precise drug delivery, and significantly enhance the effect of promoting tumor cell apoptosis. As previously reported, a block polymer composed of chitosan grafted poly-(N-3-carbobenzyloxy-l-lysine) (CZL) was used to co-transmit doxorubicin (DOX) and p53 (Lin et al., 2015). In this work, TPP as a mitochondrial targeting molecule is decorated on the surface of CZL to promote the enrichment of transmitted drugs in mitochondria.

## Materials and Methods

### Materials

CZL was synthesized in a research laboratory. 1-Ethyl-3-(3′-dimethylaminopropyl)-carbodiimide (EDC), N-hydroxysuccinimide (NHS), and curcumin were applied by Aladdin (China). HepG2 cells were provided by Chinese Academy of Sciences cell bank.

### Synthesis of TPP-CZL

Route 1 Synthesis of TPP-CZL

In this study, 43 mg TPP, 25 mg NHS, and 40 mg EDC were weighed and dissolved in 20 ml dichloromethane. Then 100 mg CZL was dissolved in 10 ml dichloromethane. After that, 20 μL triethylamine was added. Subsequently, the CZL dichloromethane solution was dripped slowly into TPP solution, and the reaction was kept for 24 h. The impurities were then removed from mixture by dialysis in water for 48 h. After dialysis, the dialysate was freeze-dried, and the TPP-CZL was obtained as a white powder with a productivity of 69%.

### Preparation of Curcumin Nanomicelles

Curcumin/TPP-CZL micelle was prepared as follows: first, 10 mg of TPP-CZL and 5.0 mg of curcumin were dissolved in 5.0 ml Dimethyl sulfoxide (DMSO) solution. Then, the solution was switched to a dialysate (molecular weight cutoff [MWCO] 3000 Da) and dialyzed in water for 48 h. Finally, the dialysate was treated with ultrasound for 5 min to disperse and filtered through a 0.45 μm filter, and the curcumin/TPP-CZL nanomicelles were obtained.

### Particle Size Distribution, Zeta Potential Measurement, and Electron Microscopic Analysis

The appropriate amount of curcumin-loaded TPP-CZL micelles was absorbed by a pipette gun and diluted by distilled water. The size of the particle and zeta potential distribution of the micelles were measured by using a zeta potential analyzer (SZ-100Z, Horiba Ltd., Japan). A small amount of curcumin/TPP-CZL micelle solution was absorbed and dripped on 200-mesh copper mesh. The solution was dried by ear ball, and then the morphology of micelles was observed under a high-resolution transmission electron microscope (JEM-2010).

### Cell Uptake

Curcumin-loaded CZL or TPP-CZL was prepared according to the preparation of the micelles as mentioned in *Preparation of Curcumin Nanomicelles*, and then the uptake of curcumin by HepG2 and L929 cells was studied. Cells were cultured in a 6-hole plate for 24 h, and then incubated with curcumin/CZL or curcumin/TPP-CZL micelles. After incubated for 6 h, the cells were harvested and washed with phosphate-buffered saline (PBS) for three times to remove curcumin on the cell surface. Curcumin was detected by flow cytometry, and the cell intake rate was calculated. The excitation wavelength was set at 425 nm, and the number of cells counted was 10000.

### Drug Loading and Releasing

Curcumin/TPP-CZL was prepared as mentioned in *Preparation of Curcumin Nanomicelles*. To detect the loading rate, the obtained curcumin/TPP-CZL was dissolved in DMSO, the concentration of curcumin was confirmed by ultraviolet spectrophotometer (Shimadzu, UV-3200), and the detected wavelength was 420 nm. The loading rate was calculated by the following equation: LC = M_1_/M_0_ × 100%, M_0_ represents the weight of TPP-CZL, and M_1_ represents the weight of loading curcumin.

Curcumin/TPP-CZL nanomicelles (containing 0.5 mg curcumin) were placed in a dialysis bag (MWCO 3000 Da). The dialysate was immersed in 20 mL PBS (pH 7.4), and the water temperature was set to 37°C. At a predetermined time point of 1, 3, 6, 9, 12, 15, 18, 24, 36, 48, and 72 h, 0.5 ml of release solution was collected, and corresponding volume of PBS was supplemented. The cumulative release rate of curcumin in micelles was calculated.

### Mitochondrial Targeting

After the curcumin loaded micelle was incubated with HepG2 cells for 3 and 6 h, the cells were removed and the mitochondria were stained with MitoTracker™ Red for 5 min, and then laser confocal microscope was used to observe the distribution of curcumin in tumor cells.

### Mitochondrial Membrane Potential

Mitochondrial membrane potential was detected by JC-1 fluorescent probe. After being treated as indicated, HepG2 cells for each group were collected and rinsed with PBS for three times. The cells were incubated with the fluorescent probe at 37°C for 30 min according to the instructions. The cells were rinsed twice with PBS and then detected by flow cytometry.

### Cytochrome C Level

HepG2 cells were treated as indicated and then the cells were fixed. Then 10 μL antibody against cytochrome C was added, and the cells were incubated in darkness for 1 h and detected by flow cytometry. Cells treated with PBS only were defined as the control group. The results were normalized with the control group.

### Bax, Bcl-2, and Caspase 3 Expression

Western blot was applied to detect the apoptosis related proteins expression. The total protein (20 μg) was separated on 12% PAGE-SDS gels. After that, they were transferred to PVDF membranes (Bio-Rad). Then, the membranes were incubated with primary antibodies against Bcl-2, Bax, and caspase 3 (1:1000) overnight at 4°C, followed by incubation with goat anti rabbit IgG-HRP antibody (1:5000) for 2 h at room temperature. The bands were detected by the enhanced chemiluminescence (ECL) system (Pierce). *β*-Actin is used as an internal parameter, and gray value is counted by ImageJ.

### Statistical Analysis

Statistical analysis was performed using software of Origin (version 8.0). Data were presented as means ± standard deviation (SD). Differences between groups were performed via Student’s t-test. *p* < 0.05 was considered to be statistically significant differences.

## Result and Discussion

**FIGURE 1:**
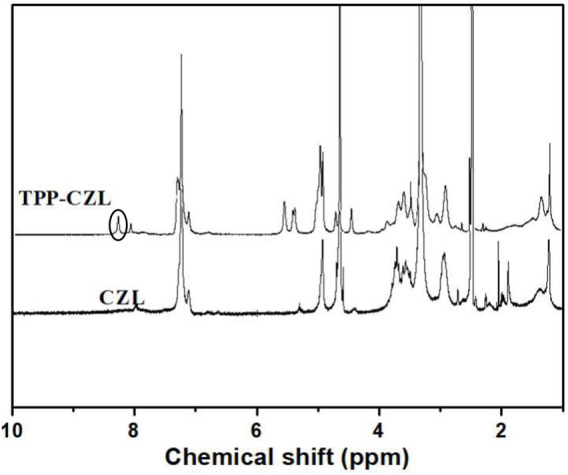
^1^H NMR spectra of CZL and TPP-CZL.

As shown in Figure 1, when compared to the spectrum of the CZL, a new peak at 8.2 has appeared, which could be attributed to the proton peak on the benzene ring of TPP (as shown in Supplementary Figure S1). From this result, it could be confirmed that TPP has been successfully connected to CZL.

**FIGURE 2:**
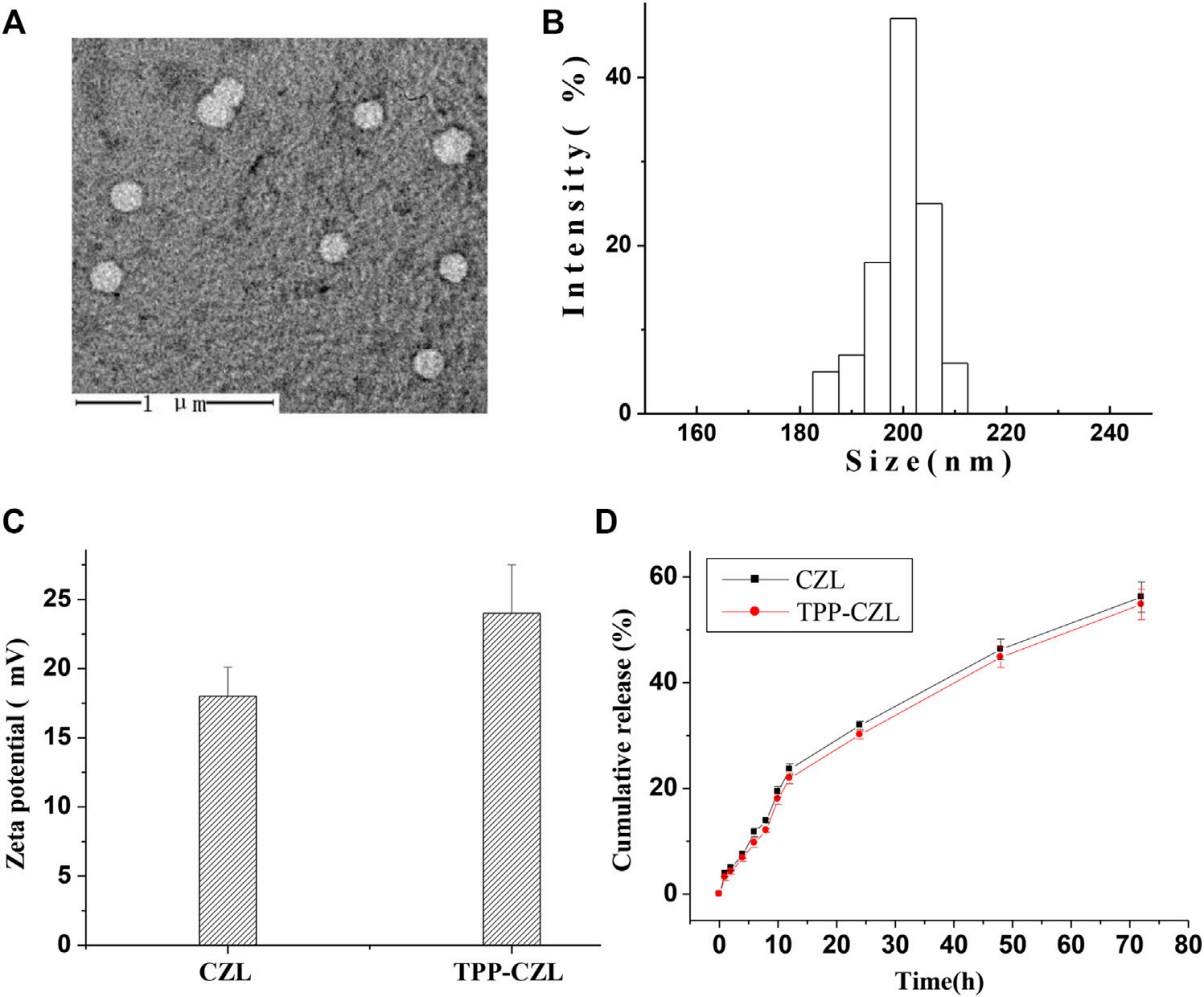
**(A)** Transmission electron microscopy (TEM) image of curcumin/TPP-CZL. **(B)** Particle size of curcumin/TPP-CZL. **(C)** Zeta potential of CZL and TPP-CZL. **(D)** Release profile of curcumin/TPP-CZL and curcumin/CZL.

It can be clearly seen from the electron microscope that the curcumin/TPP-CZL has a regular spherical shape and shows good dispersivity (Figure 2A). The particle size of curcumin/TPP-CZL nanomicelles was (208.3 ± 5.2) nm (Figure 2B), and zeta potential was (24.6 ± 3.6) MV (Figure 2C). Because of the cationic properties of TPP, the zeta potential of TPP-CZL is higher than that of CZL. Drug loading rate was 8.6 ± 1.3%, and the encapsulation rate was 73.2 ± 4.8%. Because of the positive charge of TPP, it can break through the barrier of cell membrane and mitochondrial membrane and promote the cell uptake of drugs. The literature shows that the cell membrane has a negative charge of 30–60 MV, and the inner membrane of mitochondria has a negative charge of 150–180 MV (Mahmoud et al., 2019).

Therefore, both of them can interact with the positive charge of TPP, which lead to the ability of TPP to accumulate in mitochondria being increased by 100–500 times. In this way, CZL modified by TPP can overcome the obstruction of high viscosity cell fluid and mediate the entry of curcumin into mitochondria. Both curcumin/CZL nanomicelles (containing 0.5 mg curcumin) and curcumin/TPP-CZL nanomicelles were released slowly. The release of curcumin/TPP-CZL nanomicelles was the slowest *in vitro* and had good sustained-release performance (Figure 2D).

**FIGURE 3:**
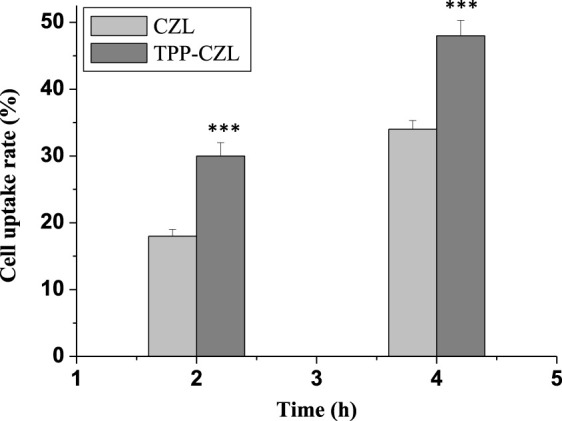
Cell uptake rate of curcumin/CZL and curcumin/TPP-CZL. *p < 0.05, **p < 0.01, ***p < 0.001 compared with CZL group.

As displayed in Figure 3, the cell uptake rate of curcumin/CZL and curcumin/TPP-CZL nanomicelles is highly time-dependent, and the cell uptake rate of curcumin/TPP-CZL nanomicelles was significantly higher than that of curcumin/CZL nanomicelles, with a significant difference. These results indicate that TPP can promote the cellular uptake of micelles. The possible reason is that the negatively charged cell membrane attracts the positively charged nanomicelles and promotes the cellular uptake of nanomicelles.

**FIGURE 4:**
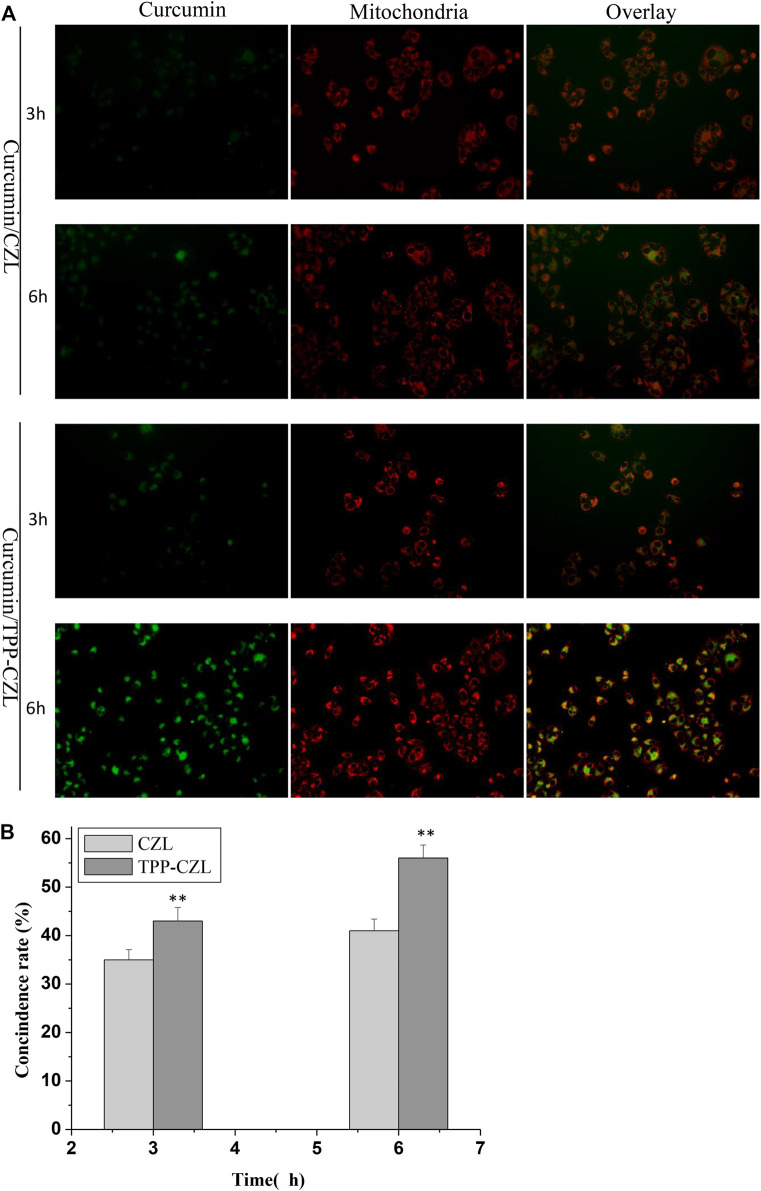
Mitochondrial targeting of CZL and TPP-CZL. *p < 0.05, **p < 0.01, ***p < 0.001 compared with CZL group.

Figure 4 illustrated that some green fluorescent emitted from nanomicelles coincide with the red fluorescent of mitochondria. After the coincidence, the color of the fluorescent became yellow, which has a good time correlation. With the extension of incubation time, the yellow gradually becomes darker. In comparison with the two groups, the distribution of TPP-CZL was significantly higher than that in CZL, indicating that TPP can promote the aggregation of micelles to mitochondria of liver cancer cells and have good mitochondrial targeting. The results of mitochondrial targeting test showed that TPP-CZL nanomicelles could well overlap with the mitochondrial site, which could perform targeted delivery of the drug to the mitochondrial site as much as possible and play a precise drug delivery effect, thus greatly improving the drug effect.

**FIGURE 5:**
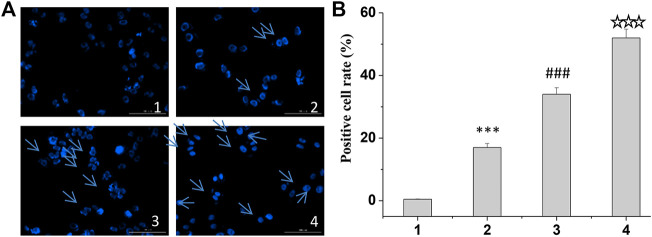
Hoechst staining of curcumin/CZL and curcumin/TPP-CZL. 1. PBS, 2. curcumin, 3. curcumin/CZL, 4. curcumin/TPP-CZL. *p < 0.05, **p < 0.01, ***p < 0.001 compared with PBS group; #*p* < 0.05, ##*p* < 0.01, ###*p* < 0.001 compared with curcumin group; *p* < 0.05, *p* < 0.01, *p* < 0.001 compared with CZL group. **(A)** Hoechst staining of cells **(B)** Positive cell rate.

Finally, according to the instructions of Hoechst 33258, the cells were stained, and the apoptosis was observed under the fluorescence electron microscope. The results showed that the cells in the treatment group became dim, the cell morphology became irregular, and some cells appeared apoptosis (Figure 5). The results showed that the positive rate of Hoechst staining was the highest in the curcumin/TPP-CZL nanomicelle group, which indicated that the curcumin/TPP-CZL nanomicelle group had the best inhibitory effect on the growth of tumor cells.

**FIGURE 6:**
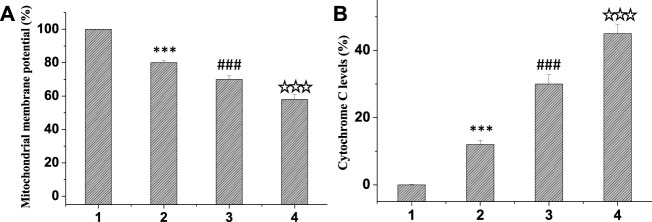
Comparison of mitochondrial membrane potential **(A)** and cytochrome C levels **(B)**. 1. PBS, 2. curcumin, 3. curcumin/CZL, 4. curcumin/TPP-CZL. *p < 0.05, **p < 0.01, ***p < 0.001 compared with PBS group; #*p* < 0.05, ##*p* < 0.01, ###*p* < 0.001 compared with curcumin group; *p* < 0.05, *p* < 0.01, *p* < 0.001 compared with CZL group.

In normal mitochondria, JC-1 aggregates in the mitochondrial matrix in the form of polymer and produces strong red fluorescence. When mitochondrial lesions occur, the mitochondrial membrane potential decreases and disappears. JC-1 could exist in the cytoplasm to form a monomer and emit green fluorescence. Therefore, JC-1 mitochondrial probe kit can quantitatively detect the proportion of diseased mitochondria according to the ratio of red and green fluorescence intensity. PBS without drug was used as the control group, and the differences among the groups were compared. Results as shown in Figure 6A, compared with the control group, the red/green fluorescence ratio of mitochondria in the treatment group was significantly reduced, and the red/green fluorescence ratio of curcumin/TPP-CZL nanomicelles group was the smallest, so the effect of promoting tumor cell apoptosis was the best.

At the beginning of apoptosis, mitochondrial outer membrane permeability increases, which promotes the release of cytochrome C into the cytoplasm, induces caspase activation cascade, and leads to cell death. The liver cancer cell line HepG2 was incubated and treated in the same group, then the cells were fixed and 10 μL cytochrome C antibody was added, incubated in dark environment for 1 h, and then detected by flow cytometry. PBS without drug was used as the control group. Figure 6B exhibited that the level of cytochrome C in the treatment group was markedly elevated compared with that in the control group. Among them, the level of cytochrome C in the curcumin/TPP-CZL nanomicelle group was the highest. Therefore, the effect of promoting tumor cell apoptosis in TPP-CZL group was the best.

**FIGURE 7:**
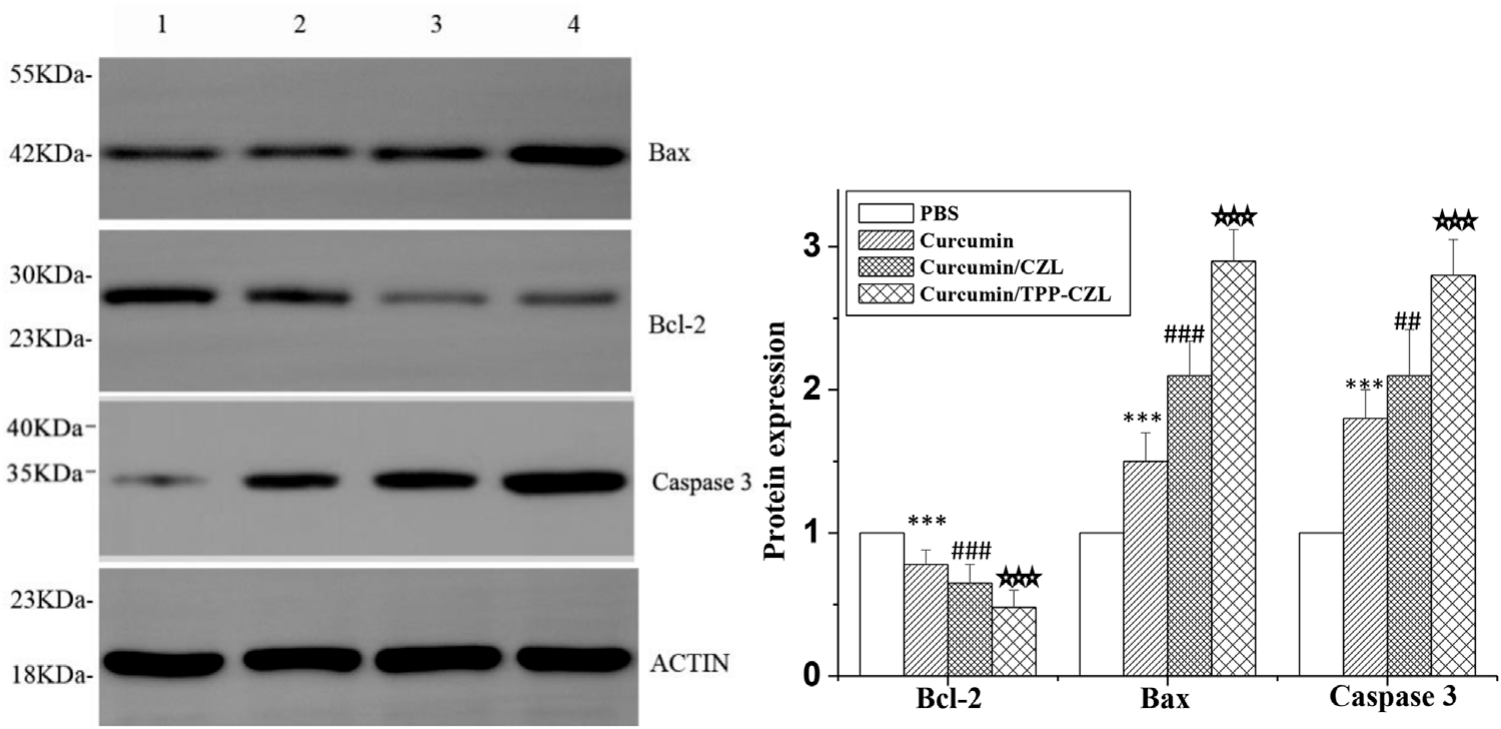
Comparison of protein expression of Bcl-2, Bax, and caspase 3.1. PBS, 2. curcumin, 3. curcumin/CZL, 4. curcumin/TPP-CZL. *p < 0.05, **p < 0.01, ***p < 0.001 compared with PBS group; #*p* < 0.05, ##*p* < 0.01, ###*p* < 0.001 compared with curcumin group; *p* < 0.05, *p* < 0.01, *p* < 0.001 compared with CZL group.

As shown in Figure 7, the level of antiapoptotic protein Bcl-2 was decreased, whereas the expressions of pro-apoptotic protein Bax and caspase-3 were observably increased in all three treated groups. Compared with the other two groups, curcumin/TPP-CZL nanomicelles group had the most obvious effect in regulating the expression of apoptosis related proteins. Apoptosis is determined by the proportion of apoptotic protein and antiapoptotic protein. The increase in the proportion of apoptotic protein and antiapoptotic protein can destroy the integrity of mitochondrial membrane, lead to the release of cytochrome c from mitochondria, activate caspase-3, and finally induce tumor cell apoptosis (Errico, 2014; Hockenbery, 2010; Wang et al., 2015; Odeh et al., 2014; Sims et al., 2014). The results showed that curcumin/TPP-CZL micelles could significantly enhance the effect of drug on tumor cell apoptosis. The results showed that curcumin/TPP-CZL nanomicelles could dramatically reduce the cellular viability, increase the percentage of Hoechst staining-positive cells, and reduce the mitochondrial membrane potential. Compared with curcumin and curcumin/CZL nanomicelles, these results showed significant differences.

## Conclusion

A mitochondrial-targeted micelle has been developed for delivering curcumin to mitochondrion of HepG2 cells. By using TPP-CZL, more curcumin can be efficiently transferred to the mitochondrion of HepG2 cells, enhancing the effect of drugs on tumor cell apoptosis.

## Data Availability

The raw data supporting the conclusion of this article will be made available by the authors, without undue reservation.
