# Supplementary material for: The Apoptosis of Liver Cancer Cells Promoted by Curcumin/TPP-CZL Nanomicelles With Mitochondrial Targeting Function
Source: Front Bioeng Biotechnol. 2022 Feb 15;10:804513. doi: 10.3389/fbioe.2022.804513 (PMC8887866; doi:10.3389/fbioe.2022.804513)
Supplement: Supplementary file 1 [file DataSheet1.docx]

**The apoptosis of liver cancer cells promoted by curcumin/TPP-CZL nanomicelles with mitochondrial targeting function**

**Supporting Information**


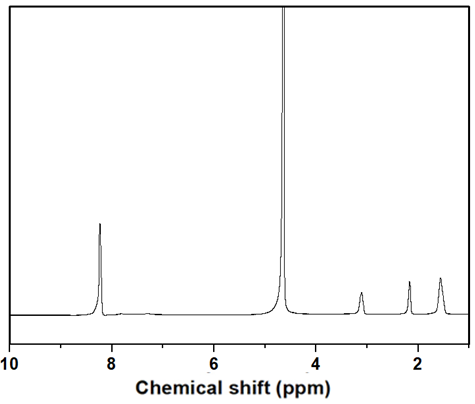


Fig.S1 ^1^H NMR spectra of TPP

***

***

Fig.S2 Cell uptake rate of curcumin/CZL and curcumin/TPP-CZL for L929 cell

***

B

A

**

**

**

Fig.S3 Mitochondrial targeting of CZL and TPP-CZL *P < 0.05, **P<0.01, ***P<0.001 compared with CZL group. A:L929 cell; B:HeLa cell.

Fig.S3 Cell viability of CZL and TPP-CZL for HepG2 *P < 0.05, **P<0.01, ***P<0.001 compared with PBS group
